# Bleeding risk in patients with multiple myeloma treated for venous thromboembolism: a MarketScan analysis

**DOI:** 10.1016/j.rpth.2022.100024

**Published:** 2022-12-23

**Authors:** Diego Adrianzen-Herrera, Pamela L. Lutsey, Katherine Giorgio, Robert F. Walker, Neil A. Zakai

**Affiliations:** 1 Department of Medicine, Larner College of Medicine at the University of Vermont, Burlington, Vermont, USA; 2 Division of Epidemiology and Community Health, University of Minnesota School of Public Health, Minneapolis, USA; 3 Department of Pathology & Laboratory Medicine, Larner College of Medicine at the University of Vermont, Burlington, Vermont, USA

**Keywords:** anticoagulants, hemorrhage, multiple myeloma, risk factors, venous thromboembolism

## Abstract

**Background:**

Multiple myeloma (MM) is associated with high risk of venous thromboembolism (VTE). Thromboprophylaxis is thoroughly studied in MM. Contrarily, studies assessing the risk of bleeding in people with MM on anticoagulation are lacking.

**Objectives:**

To determine the rate of serious bleeding in patients with MM receiving anticoagulation for VTE and the clinical factors associated with bleeding risk.

**Methods:**

Using the MarketScan commercial database, we identified 1298 people with MM treated with anticoagulation for incident VTE events between 2011 and 2019. Hospitalized bleeding was identified using the Cunningham algorithm. Rates of bleeding were calculated and Cox regression identified risk factors for bleeding.

**Results:**

Bleeding occurred in 51 (3.9%) cases during median follow-up of 1.13 years. Rate of bleeding among patients with MM on anticoagulation was 24.0 per 1000 person-years. In adjusted regression, factors associated with increased bleeding included age (HR, 1.31 per 10-year increase; 95% CI, 1.03-1.65), Charlson comorbidity index (HR, 1.29 per SD increase; 95% CI, 1.02-1.58), use of antiplatelet agents (HR, 2.4; 95% CI, 1.03-5.68), diabetes (HR, 1.85; 95% CI, 1.06-3.26), and renal disease (HR, 1.80; 95% CI, 1.05-3.16). Cumulative incidence of bleeding was 4.7%, 3.2%, and 3.4% for warfarin, low molecular weight heparin, and direct oral anticoagulants, respectively.

**Conclusion:**

In this real-world analysis, the rate of bleeding in people with MM on anticoagulation was comparable to those in other subsets of cancer-related VTE. Bleeding rate was lower with low molecular weight heparin and direct oral anticoagulants than warfarin. Higher comorbidity index, diabetes, antiplatelet agent use, and renal disease were risk factors for serious bleeding.

## Essentials

• Bleeding occurred in 3.9% patients with anticoagulated myeloma during median follow-up of 1.1 years.
• Rate of hospitalized bleeding in anticoagulated patients with myeloma was 24 per 1000 person-years.
• Bleeding rates in myeloma are comparable to other subsets of cancer-related thromboembolism.
• Clinical factors including diabetes and renal disease can identify individual bleeding risk.

## 1 Introduction

Multiple Myeloma (MM) is a plasma cell neoplasm with high risk of venous thromboembolism (VTE). [1] Disease-specific factors promote hypercoagulability, including increased plasma viscosity [2,3] and proinflammatory cytokines mediating plasma cell endothelial interactions. [4,5] The risk is accentuated by treatment-related factors including dexamethasone backbone [6] and use of immunomodulatory drugs (IMiDs), which are associated with thrombosis risk. [7,8]

Concomitantly, pathophysiology pathways in MM result in increased bleeding risk. Disease-specific factors include impaired platelet aggregation resulting from nonspecific interactions of monoclonal proteins and platelets’ surface, [3] poor coagulation triggered by M-protein interference with fibrin monomer polymerization, [4] and acquired autoantibodies against von Willebrand factor from abnormal plasma cells. [9] These mechanisms result in hemostatic abnormalities, causing altered *in vitro* platelet aggregation tests and prolonged coagulation and bleeding times. [3,4] MM treatment can also accentuate the bleeding risk, particularly via treatment-related thrombocytopenia. [1]

Given an estimated 9-fold increased risk of VTE [10] and an absolute risk over 10% without thromboprophylaxis, [11,12] research efforts focus on preventing VTE in MM, including development of risk assessment scores and consensus guidelines, [13,14] and clinical trials to define thromboprophylaxis.

In contrast, research aimed at determining the risk of bleeding in MM is scarce. This research gap is partly because of the bleeding risk poorly correlating with laboratory abnormalities, and clinically relevant bleeding being relatively rare, [15] mostly occurring in advanced stages as terminal complications. [16] Nevertheless, assessing bleeding in MM is important because management of bleeding complications is often challenging, and underestimation of bleeding risk may result in poorer outcomes. [3,9]

The risk of bleeding associated with anticoagulation for incident VTE in MM is not currently defined. As VTE incidence remains high because of poor adherence to prophylaxis [17] and higher IMiD-based therapy, [18] understanding the bleeding risk in this subgroup of cancer-related VTE constitutes a topic requiring further research. Hemostasis abnormalities inherent to MM suggest higher bleeding burden, [9] but this has not been assessed in clinical databases, and factors predicting bleeding are not documented. To address these gaps, we conducted a real-world analysis of bleeding complications in patients with MM receiving anticoagulation for VTE.

## 2 Methods

### 2.1 Data source

We used the IBM MarketScan Commercial Claims and Encounters and Medicare Supplemental databases (IBM Watson Health), from January 1, 2011 to December 31, 2019. These include healthcare claims from subjects in the U.S. with health insurance through participating programs, including most private and public insurance. These data contain individual-level information on healthcare enrollment, inpatient and outpatient visits, and prescriptions from U.S. employers, healthcare plans, and hospitals. Data are de-identified and compliant with Health Insurance Portability and Accountability act. The study was exempted by the University of Minnesota’s IRB.

### 2.2 Study population

We identified patients with MM 18 to 99 years of age with incident VTE treated with anticoagulation. MM was defined as at least 1 inpatient or 2 outpatient claims at least 7 days apart based on International Classification of Diseases (ICD)-9 or ICD-10 codes, a strategy with 88% sensitivity and 86% positive predictive value (PPV). [19] At least 90 days of continuous enrollment before VTE were required, to acquire comorbidity information. VTE was defined as at least 1 inpatient or 2 outpatient claims 7 to 184 days apart, using ICD-9 or ICD-10 codes, and initiation of anticoagulation within 30 days of diagnosis. This method has 72% sensitivity 72% and 91% PPV to identify VTE. [20] ICD codes used are summarized in Supplementary Table S1. Subjects with prior VTE during the 90-day run-in period before the incident event were excluded.

### 2.3 Outcome ascertainment

Primary outcome was severe bleeding complications, defined as any bleeding event causing hospitalization using the Cunningham algorithm. [21] This strategy has 89% to 99% PPV to identify bleeding (intracranial, gastrointestinal, and others) and can successfully identify bleeding complications from VTE anticoagulation. [22] Enrollees with hospitalized bleeding during the 90-day run-in period before VTE were excluded.

### 2.4 Prespecified covariates

Comorbidities and use of IMiDs or prescription antiplatelets (excluding aspirin) were ascertained during the time period before VTE. History of major bleeding was based on validated algorithms using ICD-9 or ICD-10 codes applied to inpatient and outpatient claims. [21,23] Charlson comorbidity index (CCI) was calculated based on comorbidity covariates. IMiD-based therapy was defined using outpatient pharmacy claims, including the National Drug Code, prescription fill date, and number of days supplied. Outpatient prescriptions were used to identify the first anticoagulant prescribed for VTE: warfarin, direct oral anticoagulants (DOACs), or low molecular weight heparin (LMWH). The validity of warfarin identification from administrative claims has 94% sensitivity and 99% PPV [24]; validity studies for DOACs and LMWH are lacking. For analysis, patients remained in the anticoagulation category initially prescribed to mimic an intention-to-treat design. [25]

### 2.5 Statistical analysis

Follow-up began at date of first anticoagulant prescription filled. Person-time at risk accumulated until incident hospitalized bleeding, death, or end of study (December 31st, 2019). Cox proportional hazard regression models estimated the hazard ratios (HRs) and 95% CIs for risk of hospitalized bleeding, adjusted for age and sex. The model comparing DOACs and LMWH to warfarin was adjusted for age, sex, CCI, and IMiD-based therapy. To identify clinical risk factors associated with bleeding, each comorbidity covariate was modeled individually, adjusted for age, sex, renal disease, and antiplatelet agent use. Proportional hazards assumption was checked with an interaction term between exposure and time. Statistical significance was determined at α < 0.05, without adjusting for multiple comparisons. Statistical analyses were performed with SAS, version 9.4 (SAS Institute Inc.).

## 3 Results and Discussion

The study population included 1298 people with established MM diagnosis and incident VTE event for which they were treated with anticoagulation. Mean age (SD) was 65.6 (12) years and 43% were female. People treated with IMiD-based regimens accounted for 54.8% and average CCI was 5.

**Figure 1:**
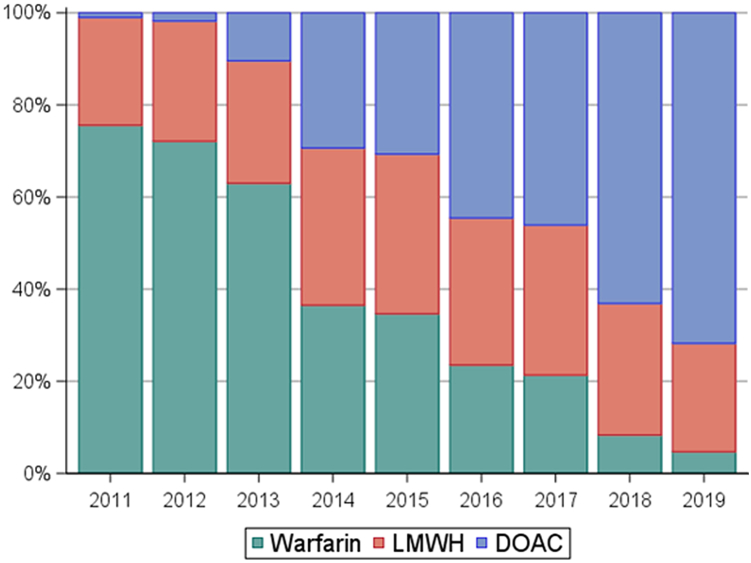
Trends in anticoagulation agents prescribed for VTE in patients with multiple myeloma from 2011 to 2019. DOAC, direct oral anticoagulant; LMWH, low molecular weight heparin; VTE, venous thromboembolism

Patient characteristics, according to anticoagulation strategy, are presented in Table 1. The agent of choice was warfarin in 598 (46.1%), LMWH in 373 (28.7%), and DOACs in 327 (25.2%) people. There were no significant differences in age or sex distribution, concomitant use of antiplatelet agents (excluding aspirin), comorbidity burden distribution, history of major bleeding, or proportion of IMiD-related VTE across the different anticoagulation strategies. There was a notable trend over time in preferred anticoagulant prescribed, favoring DOACs over warfarin later in the study period. The proportion of anticoagulants prescribed to patients with MM starting anticoagulation for VTE is shown in Figure 1.

**Table 1:** Characteristics of patients with multiple myeloma and a new thrombotic event.

Characteristic	Anticoagulation treatment		
	Warfarin	LMWH	DOAC
	(N = 598)	(N = 373)	(N = 327)
Age, mean (SD), y	67 (12)	61 (12)	67 (12)
Age ≥ 65 y, (%)	169 (28)	80 (21)	67 (20)
Sex, Female (%)	264 (44)	153 (41)	141 (43)
Comorbidities (%)			
Hypertension	437 (73)	256 (69)	222 (68)
Diabetes	175 (29)	81 (22)	82 (25)
Heart failure	140 (23)	55 (15)	66 (20)
Atrial fibrillation	73 (12)	31 (8)	42 (13)
Myocardial infarction	42 (7)	24 (6)	26 (8)
Ischemic stroke	90 (15)	37 (10)	33 (10)
Peripheral artery disease	83 (14)	49 (13)	66 (20)
Dementia	12 (2.0)	6 (1.6)	8 (2.5)
Renal disease	206 (34)	89 (24)	92 (28)
Chronic pulmonary disease	202 (34)	106 (28)	99 (30)
Liver disease	66 (11)	51 (14)	36 (11)
Depression	107 (18)	82 (22)	58 (18)
Alcohol abuse	4 (0.7)	1 (0.3)	3 (0.9)
Comorbidity index (average)	5.3 (2.6)	5.0 (2.5)	5.3 (2.7)
History of major bleeding (%)	424 (71)	267 (72)	231 (71)
Concomitant antiplatelet agent (%)^a^	31 (5)	16 (4)	19 (6)
Multiple myeloma treatment (%)^b^			
Regimen without IMiD	297 (50)	166 (45)	124 (38)
IMiD-based regimen	301 (50)	207 (56)	203 (62)

DOAC, direct oral anticoagulant; IMiD, immunomodulatory drug; LMWH, low molecular weight heparin.

a Includes: abciximab, anagrelide hydrochloride, cilostazol, dipyridamole, eptifibatide, prasugrel hydrochloride, ticagrelor, ticlopidine hydrochloride, tirofiban hydrochloride, clopidogrel hydrogen sulfate. Does not include aspirin.

b Identified at the time of diagnosis with VTE event.

With median follow-up time of 1.13 years, a total of 51 (3.9%) hospitalized bleeding events occurred in patients with MM treated for VTE. The overall incidence rate of hospitalized bleeding in this population was 24 per 1000 person-years.

Of the bleeding events, 28 occurred among warfarin users, 12 among LMWH users, and 11 among DOACs users. The cumulative incidence of hospitalized bleeding was 4.7% for patients treated with warfarin, 3.2% for those treated with LMWH, and 3.4% for those treated with DOACs. However, these differences were not statistically significant, and estimated incidence rates were similar across anticoagulant agents: 25.7 per 1000 person-years for warfarin, 19.5 per 1000 person-years for LMWH, and 26.3 per 1000 person-years for DOACs. In analyses adjusted for age, sex, CCI, and IMiDs use, the HR of hospitalized bleeding associated with LMWH was 0.90 (95% CI, 0.45-1.82), whereas for DOACs it was 0.97 (95% CI, 0.48-1.96), as compared with warfarin.

In adjusted analysis, clinical risk factors significantly associated with risk of hospitalized bleeding included older age (HR, 1.26 per 10-year increase; 95% CI, 1.01-1.58), higher CCI (HR, 1.29 per SD increment; 95% CI, 1.02-1.58), use of antiplatelet agents (HR, 2.4; 95% CI, 1.03-5.68), diabetes (HR, 1.85; 95% CI, 1.06-3.26), and renal disease (HR, 1.80; 95% CI, 1.05-3.16). Previous history of major bleeding and use of IMiD for MM treatment, did not significantly influence the risk of hospitalized bleeding (Table 2).

**Table 2:** Risk factors for major bleeding in patients with multiple myeloma treated for VTE.

Characteristic	Outcome frequency		Adjusted analysis	
	N	%	HR	(95% CI)
Anticoagulation agent				
Warfarin	28	4.7	Ref	
LMWH	12	3.2	0.95	(0.47-1.93)
DOAC	11	3.4	1.01	(0.50-2.04)
Sex, female	20	3.6	0.84	(0.48-1.47)
Age (per 10-y increase)			1.26	(1.01-1.58)
Comorbidity score (per 1 SD increase)			1.29	(1.02-1.58)
Antiplatelet agent use (excludes aspirin)^a^	66	5.1	2.40	(1.03-5.68)
History of major bleeding	36	3.9	1.00	(0.50-1.70)
Multiple myeloma treatment^b^				
Regimen without IMiD	28	4.8	Ref	
IMiD-based regimen	23	3.2	0.68	(0.39-1.17)
Comorbidities^c^				
Hypertension	42	4.6	1.60	(0.75-3.41)
Diabetes	22	6.5	1.85	(1.06-3.26)
Heart failure	16	6.1	1.46	(0.77-2.75)
Atrial fibrillation	9	6.2	1.80	(0.86-3.75)
Myocardial infarction	4	4.4	1.10	(0.38-2.36)
Ischemic stroke	10	6.3	1.16	(0.56-2.39)
Peripheral artery disease	8	4.0	0.84	(0.39-1.84)
Dementia	0	-	-	-
Renal disease	23	5.9	1.80	(1.05-3.16)
Chronic pulmonary disease	24	5.9	1.60	(0.97-2.81)
Liver disease	6	3.9	1.22	(0.51-2.81)
Depression	11	4.5	1.33	(0.68-2.60)
Alcohol abuse	0	-	-	-

DOAC, direct oral anticoagulant; IMiD, immunomodulatory drug; HR, hazard ratio; LMWH, low molecular weight heparin; VTE, venous thromboembolism.

a Includes: abciximab, anagrelide hydrochloride, cilostazol, dipyridamole, eptifibatide, prasugrel hydrochloride, ticagrelor, ticlopidine hydrochloride, tirofiban hydrochloride, clopidogrel hydrogen sulfate. Does not include aspirin.

b Identified at the time of diagnosis with VTE event.

c Analysis adjusted for age, sex, antiplatelet agent use, and renal disease.

Bleeding is an important complication of VTE treatment and bleeding risk from anticoagulants is especially high in people with cancer. Patients with MM constitute a unique group with disproportionately high VTE risk and concomitant hemostatic abnormalities which may result in greater bleeding. [3,4] In this real-world population of patients with MM who received anticoagulation for incident VTE, the rate of severe bleeding complications, defined as hospitalized bleeding, was found to be comparable to those reported in other subsets of cancer-related VTE. [26] Balancing the increased and competing risk of bleeding in people with hematologic malignancies and thrombosis can be difficult, but our results suggest no disparate bleeding complications in MM versus those reported with other cancers.

Despite considerable efforts to define thromboprophylaxis strategies in MM, VTE remains a major burden, with both real-world and clinical trial results showing over 10% incidence of VTE. [27,28] Therefore, it is important to assess the safety profile of different anticoagulation choices in this population. Our results show that the risk of hospitalized bleeding was similar across all anticoagulation strategies. The point incidence of hospitalized bleeding was lower in patients treated with DOACs or LMWH compared with warfarin, though differences were not statistically significant and estimated bleeding rates were similar for all agents.

Our analysis also reveals a significant trend in the preferred anticoagulation agent for patients with MM. We found a remarkable decrease in warfarin prescriptions over the past decade in favor of DOACs, which were the preferred agent in over 75% of MM patients with incident VTE in the last year of analysis (2019). Although warfarin falling out of favor would not be entirely surprising, this trend could represent clinician preference for DOACs over LMWH as antithrombotic drugs in MM. However, these findings could also be explained by changes in availability of antithrombotic agents over time, independent of clinician preference. Further studies are needed to assess clinician predilections, which could also inform VTE treatment or prevention guidelines.

Given that the risk was similar for all anticoagulation strategies, the choice of VTE treatment could be informed by the individual’s risk of bleeding. In our cohort, we found that increasing age, higher CCI, concomitant antiplatelet agents, diabetes, and renal disease were factors significantly associated with increased risk of hospitalized bleeding in patients with MM undergoing anticoagulation for VTE. IMiDs-based therapy preceding a VTE event had no association with bleeding risk. Although there are several risk scores aimed at predicting VTE in MM, [29] risk models for serious bleeding complications in this population are lacking. These clinical risk factors may be included in risk assessment models to help clinicians identify a selective group of patients with MM with increased individual bleeding risk from anticoagulation.

We acknowledge important limitations of this study. First, the possibility of misclassification of conditions of interest; a concern associated with all administrative claim analyses, particularly, underestimation of bleeding resulting from limiting severe events to those requiring hospitalization. Nevertheless, the algorithms utilized to identify MM, VTE, hospitalized bleeding, and anticoagulant prescriptions all have high PPV. Although not yet quantified, algorithms to identify DOACs and LMWH are expected to be as accurate as those for warfarin. Furthermore, the bleeding rates in our cohort seem consistent with the limited data available on bleeding risk among patients with MM on therapeutic anticoagulant doses. [29,30] Second, the possibility that uncontrolled confounding explains our findings. Given the nonrandomized design, causal inference is limited. However, the clinical risk factors we described are similar to those reported in other cancer-related VTE populations. [26] Third, the circumstances within which individual bleeding events occurred could not assessed as MM-specific factors were not available in the database. Missing information includes survival, disease status (new diagnosis or relapsed), use of thromboprophylaxis, coexisting thrombocytopenia, or stem cell transplantation status. These factors can substantially affect bleeding risk and their inclusion in future studies is necessary to best identify the clinical settings with highest bleeding risk. Fourth, aspirin use was not available as administrative claims cannot reliably account for medications available over the counter. Lastly, precision was poor for some analyses, and we were unable to evaluate individual DOACs.

Despite the limitations, our results show that severe bleeding complications of VTE anticoagulation among people with MM are not different than those reported in other cancer types, with an estimated rate of hospitalized bleeding that was similar across all anticoagulant agents. Clinicians should focus on identifying clinical risk factors to assess individual bleeding risk.
