# Supplementary material for: Bleeding risk in patients with multiple myeloma treated for venous thromboembolism: a MarketScan analysis
Source: Res Pract Thromb Haemost. 2022 Dec 23;7(1):100024. doi: 10.1016/j.rpth.2022.100024 (PMC9982328; doi:10.1016/j.rpth.2022.100024)
Supplement: Supplementary Table S1 [file mmc1.docx]

Supplemental Table 1.

Claim codes utilized in cohort inclusion criteria

| **Disease/Condition** | **ICD-9 codes** | **ICD-10 codes** |
| --- | --- | --- |
| Incident VTE | 415.1, 451.1, 453.2, 453.4, 453.9, 453.82, 453.83, 453.84, 453.85, 453.86, 453.87, 453.89 | I26, I80.1, I82.4, I80.20, I80.22, I80.23, I80.29, I82.60, I82.62, I82.A1, I82.B1, I82.C1, I82.220, I82.210, I82.290, I82.890 |
| Prevalent Multiple Myeloma | 203.00, 203.01, 203.10, 203.11, 203.12 | C90 |

ICD: International Classification of Diseases, VTE: Venous Thromboembolism.
